# KLIKK proteases of *Tannerella forsythia*: putative virulence factors with a unique domain structure

**DOI:** 10.3389/fmicb.2015.00312

**Published:** 2015-04-21

**Authors:** Miroslaw Ksiazek, Danuta Mizgalska, Sigrum Eick, Ida B. Thøgersen, Jan J. Enghild, Jan Potempa

**Affiliations:** ^1^Department of Microbiology, Faculty of Biochemistry, Biophysics and Biotechnology, Jagiellonian UniversityKrakow, Poland; ^2^Laboratory of Oral Microbiology, Department of Periodontology, University of BernBern, Switzerland; ^3^Department of Molecular Biology and Genetics, Center for Insoluble Protein Structures (inSPIN) and Interdisciplinary Nanoscience Center (iNANO), Aarhus UniversityAarhus, Denmark; ^4^Department of Oral Immunology and Infectious Disease, University of Louisville School of DentistryLouisville, KY, USA

**Keywords:** infectious disease, periodontal disease, *Tannerella forsythia*, proteases, virulence

## Abstract

Comparative genomics of virulent *Tannerella forsythia* ATCC 43037 and a close health-associated relative, *Tannerella BU063*, revealed, in the latter, the absence of an entire array of genes encoding putative secretory proteases that possess a nearly identical C-terminal domain (CTD) that ends with a -Lys-Leu-Ile-Lys-Lys motif. This observation suggests that these proteins, referred to as KLIKK proteases, may function as virulence factors. Re-sequencing of the loci of the *KLIKK* proteases found only six genes grouped in two clusters. All six genes were expressed by *T. forsythia* in routine culture conditions, although at different levels. More importantly, a transcript of each gene was detected in gingival crevicular fluid (GCF) from periodontitis sites infected with *T. forsythia* indicating that the proteases are expressed *in vivo*. In each protein, a protease domain was flanked by a unique N-terminal profragment and a C-terminal extension ending with the CTD. Partially purified recombinant proteases showed variable levels of proteolytic activity in zymography gels and toward protein substrates, including collagen, gelatin, elastin, and casein. Taken together, these results indicate that the pathogenic strain of *T. forsythia* secretes active proteases capable of degrading an array of host proteins, which likely represents an important pathogenic feature of this bacterium.

## Introduction

*Tannerella forsythia* is a Gram-negative, asaccharolytic bacterium residing in the oral cavity. Its primary niche is a subgingival space below the gum line where, together with a diverse community of microorganism, it is a part of subgingival bacterial plaque (Colombo et al., 2009). The plaque becomes pathogenic when *T. forsythia* is joined by *Porphyromonas gingivalis* and *Treponema denticola*, grouped together into the so-called “red complex” of oral bacteria. Through release of various virulence factors, these bacteria disturb homeostasis in the periodontium leading to a sustained host inflammatory response, which erodes tooth-supporting tissues (Schenkein, 2006). Disease progression is manifested by attachment loss, alveolar bone resorption, and formation of deep periodontal pockets. Severe cases of periodontitis, which inflict 7–15% of the human population, may eventually result in tooth loss, if untreated (Socransky et al., 1998; Tanner and Izard, 2006; Colombo et al., 2009; Montagner et al., 2012). In addition, periodontal infection is associated with systemic diseases such as cardiovascular disease, rheumatoid arthritis, and diabetes (Suzuki et al., 2010; Lalla and Papapanou, 2011; Jeftha and Holmes, 2013; Koziel et al., 2014).

The common feature of the red complex bacteria is secretion of proteolytic enzymes, which are established virulence factors of *P. gingivalis* and *T. denticola* (Imamura et al., 2003; Ishihara, 2010). *P. gingivalis* proteases are especially well-investigated with respect to their structure and housekeeping functions, as well as their role in deterring host defenses and fuelling local inflammation (Guo et al., 2010). By stark contrast, very little is known regarding the *T. forsythia* proteases (Saito et al., 1997; Sharma, 2010), and only three enzymes have been characterized to date. The cysteine protease PrtH possesses a predicted caspase-like fold and detaches adherent cells from the substratum and stimulates IL-8 expression (Nakajima et al., 2006; Tomi et al., 2008; Pei and Grishin, 2009). PrtH levels are correlated with periodontal attachment loss (Hamlet et al., 2008). A second protease, karilysin, is structurally related to human matrix metalloproteases (Karim et al., 2010; Cerdà-Costa et al., 2011; Guevara et al., 2013). The enzyme may contribute to *T. forsythia* virulence by shedding of soluble, fully active tumor necrosis factor α(TNFα) from the macrophage surface (Bryzek et al., 2014), inhibition of all pathways of the complement system (Jusko et al., 2012), and degradation of the antimicrobial peptide LL-37 (Koziel et al., 2010), a component of innate immunity essential for periodontium homeostasis (Eick et al., 2014). The third protease is mirolase, a calcium-dependent serine protease with a unique mechanism of activation that may contribute to *T. forsythia* virulence by hydrolysis of human fibrinogen, hemoglobin, and LL-37 (Ksiazek et al., 2015a).

*T. forsythia* karilysin is a unique multi-domain protein encompassing a typical N-terminal signal peptide (SP), followed by a short propeptide conferring latency on the secreted protease with the fold closely resembling an animal matrix metalloproteinase-like catalytic domain (CD), and a C-terminal extension (CTE) (Karim et al., 2010; Cerdà-Costa et al., 2011; Lopez-Pelegrin et al., 2015). The most C-terminal part of the CTE shares sequence similarity with a conserved CTD that serves as a signal to translocate secreted proteins across the bacterial outer membrane via a novel type 9 secretion system (T9SS) first described in *P. gingivalis* (Sato et al., 2005; Nguyen et al., 2007, 2009). This system also operates in *T. forsythia* and another periodontal pathogen, *Prevotella intermedia*, mediating secretion of major virulence factors in all these pathogenic species. These virulence factors include gingipains of *P. gingivalis*, interpain of *P. intermedia*, and surface layer proteins as well as the leucine-rich protein BspA of *T. forsythia* (Sato et al., 2013; Zhou et al., 2013; Narita et al., 2014; Tomek et al., 2014).

The primary structure of the karilysin-derived CTD deviates from that of the classical CTD; nevertheless, karilysin secretion is dependent on T9SS (Narita et al., 2014). A blast analysis of a putative *T. forsythia* transcriptome revealed the presence of nine proteins with a KLIKK CTD, as in karilysin. Eight of these putative proteins contain a protease domain; thus we refer to them as the KLIKK proteases. They are clustered in three *loci* in *T. forsythia* ATCC 43037 but are absent in the periodontal health-associated *Tannerella BU063* (Oral Taxon 286) (Beall et al., 2014). This observation suggests the KLIKK proteases may be important virulence factors. Therefore, to verify the presence, structure, expression, and activity of these putative proteases, we re-sequenced a part of the *T. forsythia* genome, determined the level of expression of the KLIKK proteases *in vitro* and *in vivo*, and characterized their proteolytic activity.

## Materials and methods

### Chemicals and reagents

The restriction endonucleases BamHI and XhoI, T4 DNA ligase, dNTPs, GeneJET™ Gel Extraction Kit, GeneJET™ PCR Purification Kit, and GeneJET™ Plasmid Miniprep Kit were purchased from Thermo Scientific Fermentas (Vilnius, Lithuania). Phusion DNA Polymerase was obtained from Thermo Scientific Finnzyme (Woburn, MA, USA). The QuikChange Lightning Site-Directed Mutagenesis Kit was obtained from Stratagene (La Jolla, CA, USA). All primers used in the study were synthesized by Genomed and “Pracownia Sekwencjonowania DNA i Syntezy Oligonukleotydów” IBB PAN (Warsaw, Poland). The expression vector pGEX-6P-1, glutathione-Sepharose 4 Fast Flow and 3C protease (PreScission) were purchased from GE Healthcare Life Sciences (Little Chalfont, UK). FTC-casein and Protein Concentrators (9K MWCO, 7 mL) were obtained from Pierce Thermo Fisher Scientific (Rockford, IL). Azocoll was purchased from Calbiochem Merck (Darmstadt, Germany), DQ-gelatin was purchased from Life Technologies Thermo Fisher Scientific (Rockford, IL USA), and Elastin Congo Red was purchased from Sigma (St. Louis, MO). The molecular weight marker: “LMW” (molecular mass range: 14–97 kDa) was purchased from GE Healthcare. Unless otherwise indicated, all other chemicals were obtained from BioShop Canada (Burlington, ON, Canada).

### Re-sequencing of loci encoding putative proteases with the KLIKK ending

PCR was performed using a CFX96 Touch machine (Bio-Rad Life Science Research, Hercules, CA). Each reaction consisted of 10 μl of 5 × Phusion HF Buffer, 5 μl of 2 mM dNTPs, 5 μl of specific primer mix, as listed in Supplementary Table 1 (final primer concentration, 0.5 μM), 1.5 μl of DMSO, 2.5 μl (50 ng) of genomic DNA isolated from *T. forsythia* strain ATCC 43037, 1 μl of Phusion DNA Polymerase, and water to a final volume of 50 μl. The PCR reaction consisted of an initial denaturation step at 98°C for 2 min, followed by 35 cycles of 10 s at 98°C, 30 s at a primer specific annealing temperature (Supplementary Table 1), and 30 s per 1 kb of predicted PCR product at 72°C, and a final extension step (10 min at 72°C). Amplicons were separated by electrophoresis in a 1% agarose gel, cut from the gel, and purified using the GeneJET™ Gel Extraction Kit. The purified PCR products were sent for DNA sequencing using specific primers (Supplementary Table 1).

### Molecular cloning

Genomic DNA was isolated from *T. forsythia* strain ATCC 43037 using the Genomic Mini System (A&A Biotechnology, Gdansk, Poland), according to the manufacturer's recommendations. The entire genes encoding forsilysin (*BFO_1168*), miropsin-1 (*BFO_1179*), mirolysin (*BFO_2661*), and miropsin-2 (*BFO_2679*), except for the nucleotide sequences encoding the predicted SPs, were amplified by PCR, purified, and cloned into the pGEX-6P-1 expression vector using BamHI/XhoI and BamHI/EcoRI sites, and specific PCR primers (Supplementary Table 1). The plasmids encoding karilysin and mirolase were obtained as described previously (Karim et al., 2010; Ksiazek et al., 2015a). The resulting recombinant products include an N-terminal GST tag and a PreScission protease cleavage site, followed by the protein sequence. All plasmids were verified by DNA sequencing.

### Real-time PCR

*T. forsythia* RNA was isolated from 5-day-old plates using an innuPREP RNA Mini Kit (Analytic Jena, Jena, Germany). Before cDNA synthesis, the RNA was digested with RQ1 DNase (Promega, Madison, WI, USA) and purified using the TRI Reagent (Ambion, Life Technologies). RNA (1.6 μg) was then reverse transcribed with cDNA High Capacity cDNA Reverse Transcription Kit (Applied Biosystems, Life Technologies). The real-time PCR was performed on a CFX96 Touch machine. A single reaction consisted of 7.5 μl of FastStart Essential DNA Green Master mix (Roche, Basel, Switzerland), 1 μl of 300 nM target specific primer mix (Supplementary Table 1), 5 μl of cDNA (diluted 1:10), and 1.5 μl of water. The PCR reaction consisted of an initial denaturation step at 95°C for 10 min, followed by 40 cycles of 10 s at 95°C, 30 s at a primer specific annealing temperature, and 30 s at 72°C. All samples were analyzed in triplicate. Relative transcripts levels were calculated using the modified ΔΔCt method (Pfaffl, 2001).

### Determination of the expression of the KLIKK proteases *in vivo*

Patients with diagnosed chronic periodontitis attending the Clinic of Periodontology at the University Hospital of Jena were recruited for this study. For detection of *T. forsythia* in clinical samples, GCF samples were obtained from patients with severe periodontitis [aggressive periodontitis (*n* = 17) and chronic periodontitis (*n* = 37)] and from six healthy controls. Two paper points were inserted in each pocket for 20 s, and DNA was subsequently extracted using the Genomic Mini System, according to the manufacturer's recommendations. PCR for detection of *T. forsythia* was carried out as described previously (Ashimoto et al., 1996). To determine whether KLIKK protease genes were transcribed *in vivo*, an aliquot of GCF was stored at −20°C until mRNA was extracted for RT-PCR analysis. Total RNA from ~ 50 μl of GCF was purified using an RNeasy kit (Qiagen, Venlo, Limburg, Netherlands), and cDNA was synthesized from 1 μg of total RNA employing the Omniscript kit (Qiagen). Oligonucleotide primers (Supplementary Table 1) were used at a final concentration of 0.5 μM. The PCR with Taq polymerase was performed for 30 cycles, consisting of denaturation at 94°C for 30 s, annealing at 56°C for 25 s, and polymerization at 72°C for 30 s. The amplified PCR products were then analyzed by electrophoresis in a 2% agarose gel.

### Expression and purification of recombinant proteins

The plasmids encoding recombinant *T. forsythia* KLIKK proteases were transformed into *Escherichia coli* strain BL21 (DE3) (New England Biolabs, Ipswich, MA) under the control of the T7 promoter. Transformed *E. coli* hosts were grown in LB medium at 37°C to an OD_600_ ranging from 0.75 to 1 and cooled for 30 min at 4°C, and expression of recombinant proteins was induced by the addition of 0.25 mM isopropyl-1-thio-β-D-galactopyranoside (IPTG). After culture for 6 h at 20°C, cells were harvested by centrifugation (15 min, 6000 × g, 4°C), re-suspended in PBS (15 ml per pellet from 1 L of culture), and subsequently lysed by sonication (cycle of 30 × 0.5 s pulses at a power output of 70% per pellet from 1 L of culture) using a Branson Sonifier Digital 450 (Branson Ultrasonics, Danbury, CT, USA). The cell lysates were clarified by centrifugation (40 min, 40,000 × g, 4°C), filtered through a 0.45 mm syringe filter, and loaded onto a glutathione-Sepharose 4 Fast Flow column (bed volume, 5 ml) equilibrated with PBS at 4°C. Recombinant proteins were eluted using 50 mM Tris-HCl, pH 8.0, supplemented with 10 mM reduced glutathione. Alternatively, 10 ml of PBS containing 100 μl of PreScission protease stock solution (1 U ml^−1^) was applied to the column and incubated for 40 h at 4°C. Protein concentration was determined by measurement of absorbance at 280 nm using a Nanodrop spectrophotometer (NanoDrop products, Wilmington, DE, USA). The purity of the proteins was verified by SDS-PAGE electrophoresis using 10% gels (acrylamide/bis-acrylamide ratio, 33:1) and the Tris-HCl/Tricine buffer system (Schägger and von Jagow, 1987). Gels were stained with 0.1% Coomassie Brilliant Blue R-250 in 10% acetic acid and destained in 30% methanol, 10% acetic acid, and 1% acetic acid.

### Zymography

Zymographic analysis was performed on the purified KLIKK proteases, mixed 1:1 with sample buffer (0.125 M Tris-HCl, pH 7.8, 20% glycerol, 4% SDS, and 0.1% Bromophenol Blue) for 15 min at 20°C, and then electrophoretically resolved in 12% SDS-PAGE gels (acrylamide/bis-acrylamide ratio, 27.5:1) containing casein or gelatin at a final concentration of 0.1 mg ml^−1^. The gels were washed twice in 2.5% Triton X-100 for 30 min, followed by incubation in developing buffer (0.2 M Tris-HCl, pH 7.8, 5 mM CaCl_2_, and 1 mM DTT) for 3 h at 37°C. Finally, gels were incubated in destaining/fixing solution [methanol:acetic acid:water (30:10:60)], then stained with 0.1% amido black in 10% acetic acid for 1 h and destained successively in destaining/fixing solution, 10% acetic acid, and 1% acetic acid, which revealed clear zones of substrate hydrolysis on a blue background.

### Proteolytic activity assay

To determine the activity of the proteases against Azocoll and Elastin Congo Red, 2.5 μg of each KLIKK protease was diluted in assay buffer (50 mM Tris, pH 8.0, 2.5 mM CaCl_2_, and 0.02% NaN_3_) to a final volume of 125 μl, and then mixed with 125 μl of substrate in assay buffer (15 mg ml^−1^), followed by incubation for 2 h (Azocoll) or 16 h (Elastin Congo Red) at 37°C with shaking. For Elastin Congo Red, human neutrophil elastase (BioCentrum, Krakow, Poland) was used as a positive control. Undigested substrate was removed by centrifugation (5 min, 16,100 × g), and the absorbance of the supernatant at 520 nm (Azocoll) or 495 nm (Elastin Congo Red) was measured using a SpectraMAX microplate reader (Molecular Devices, Sunnyvale, CA). For FTC-casein and DQ-gelatin, mixtures containing 2.5 μg of each KLIKK protease in 100 μl of assay buffer were prepared directly in the wells of black microtiter plates (Nunc, Roskilde, Denmark). Next, 100 μl of substrate solution in assay buffer (50 and 100 μg ml^−1^ for FTC-casein and DQ-gelatin, respectively) was added, and the rate of substrate hydrolysis was recorded as the increase in fluorescence (λ_ex_ = 385 nm, λ _em_ = 438 nm for FTC-casein, and λ_ex_ = 495 nm, λ _em_ = 515 nm for DQ-gelatin) using a fluorescence microplate reader (SpectraMaxGmini XS, Molecular Devices).

## Results

### Re-sequencing of fragments of T. forsythia genome containing KLIKK proteases

**Figure 1:**
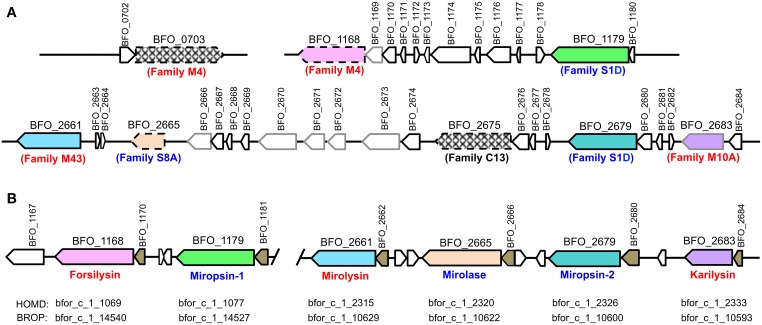
**Gene arrangement of *T. forsythia* ATCC 43037 KLIKK proteases. (A)** A GenBank (accession number: CP003191, http://www.ncbi.nlm.nih.gov/genbank) deposited sequence, prior to re-sequencing. Arrows framed by a gray line—ORFs identified as pseudogenes; hatched and color arrows framed with a broken line - protease genes absent in the genome and with errors in the prediction of ORF N-termini, respectively. **(B)** Re-sequenced and corrected gene arrangement. Arrow boxes: black, genes; gray, pseudogenes; color filled, genes encoding proteases; and brown, genes encoding putative lipoproteins. Designations of the peptidase family (M, metallopeptidase; S, serine peptidase; and C, cysteine peptidase) of putative proteases are indicated below the arrows on **(A)**. Names of the proteases characterized with respect to expression by *T. forsythia* and proteolytic activity is shown in **(B)**. Red, metalloproteases; blue, serine proteases. HOMD: Human Oral Microbiome Taxon Description, *Tannerella forsythia* strain 92A2 (HOMD, http://www.homd.org); BROP: Bioinformatics Resource for Oral Pathogens, *Tannerella forsythia* 92A2 (BROP, http://www.brop.org). These sequence data have been submitted to the GenBank database under accession numbers KP715368 and KP715369.

The sequence of karilysin and mirolase available in the database (GenBank database, accession number: CP003191) was previously shown to be incorrect (Karim et al., 2010; Ksiazek et al., 2015a). Thus, we re-sequenced three fragments of the *T. forsythia* genome containing the eight KLIKK protease *loci* (Figure 1A). Amplification of the desired regions was performed with primers nested in genes flanking the protease open reading frames (ORFs). Six KLIKK proteases were found: three serine proteases (*BFO_1179*, miropsin-1; *BFO_2665*, mirolase; *BFO_2679*, miropsin-2) and three metalloproteases (*BFO_1168*, forsilysin; *BFO_2661*, mirolysin; *BFO_2683*, karilysin) (Figure 1B). The sequencing analysis revealed that the available genomic sequence of *T. forsythia* contains a number of errors, including the presence of non-existing ORFs (*BFO_0703* and *BFO_2675*), errors in the prediction of ORF N-termini (forsilysin, mirolase), and differences in amino acid sequences within ORFs resulting from point mutations, deletions, and insertions. It should be noted that only the database sequence for mirolysin is correct.

To exclude the possibility that the two ORFs (*BFO_0703* and *BFO_2675*) we did not find within the analyzed fragments of the *T. forsythia* genome were present at other loci, we tried to amplify these two genes (using primers designed on the sequences in the database), but the results were negative in both cases, and we failed to obtain the desired PCR products.

All the KLIKK proteases were preceded by ORFs encoding small (360–550 bp) putative proteins, predicted to be lipoproteins of unknown function (Figure 1B). Alignment of amino acid and nucleic acid sequences of these six ORFs revealed that they share no significant sequence similarity. These ORFs possess a common feature: at the end of each predicted lipoprotein there is a ~50 bp long AT rich tract, but without any consensus sequence (data not shown).

### Multi-domain structure of the KLIKK proteases

**Figure 2:**
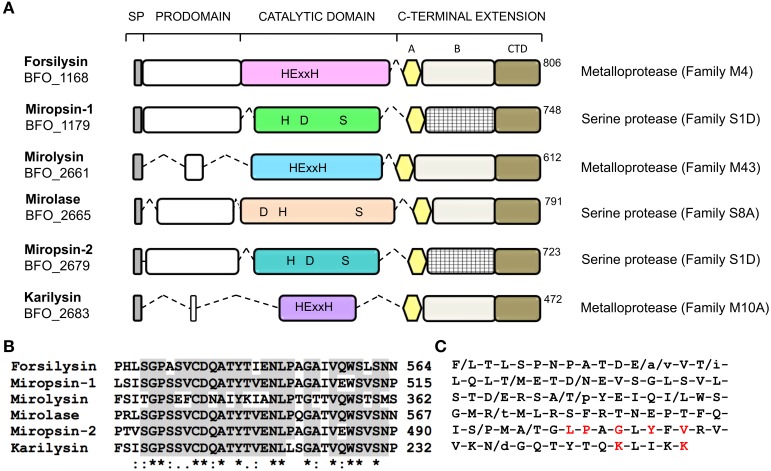
**Predicted multi-domain structure of *T. forsythia* KLIKK proteases. (A)** The corrected sequences of *T. forsythia* proteases were aligned using ClustalW (http://www.ebi.ac.uk/Tools/msa/clustalw2/). Based on this, the following structure of KLIKK proteases, beginning from the N-terminus, was proposed: SP (gray box), NTP (white box), CD (color boxes) containing all amino acids directly involved in proteolysis (in black), and CTE containing the variable region B flanked by two conserved regions, 30 amino acids A, and the last 86 amino acids (CTD). The catalytic amino acids and protease family (in brackets next to the gene name) were predicted using the MEROPS database. The signal peptides were predicted using the SignalP 3.0 Server (Bendtsen et al., 2004). **(B)** Alignment of region A from KLIKK proteases. The identical amino acids in at least four proteases are highlighted in gray, and the asterisks indicate conserved amino acids in all proteases. **(C)** Consensus sequence of CTD from KLIKK proteases. Low case fonts indicate residues occurring only once at the specific position. The amino acids conserved in known CTDs of proteins of *P. gingivalis* and *T. forsythia* is marked in red.

The alignment of the KLIKK proteases revealed the multi-domain structure of the analyzed enzymes (Figure 2). Beginning from the N-terminus, the KLIKK proteases consist of a classical SP (predicted using SignalP 3.0 Server, Bendtsen et al., 2004), an N-terminal profragment (NTP), a CD containing all the amino acid residues crucial for proteolytic activity (MEROPS database, Rawlings et al., 2014), and a CTE. The CTE is unique for each protease and consists of a sequence of 140–160 residues flanked by two conserved regions: a motif of 30 residues at the beginning and a domain of 86 residues at the end. The variable region of the CTE was identical only for the two S1D serine proteases, miropsin-1 and miropsin-2. Interestingly, with the exception of the CTD domain, which shares some similarity with classical CTDs found in proteins of *P. gingivalis* and *T. forsythia*, e.g., gingipains and *T forsythia* S-layer proteins, respectively, other segments of the KLIKK proteases flanking the protease domains do not possess significant homology to any known proteins (Nguyen et al., 2007).

### Expression level and prevalence of KLIKK proteases

**Figure 3:**
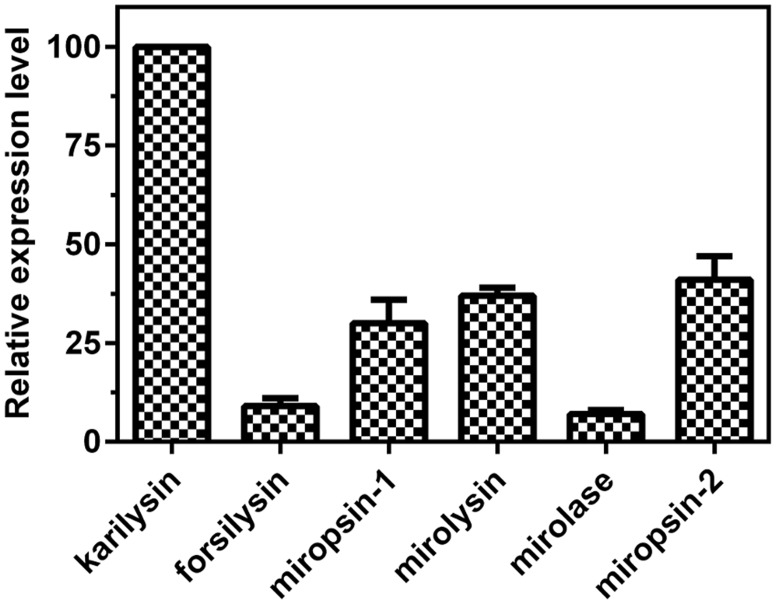
**The expression level of KLIKK proteases found in the genome of *T. forsythia***. The expression level of six proteases was determined by real-time PCR. The transcript level of karilysin was arbitrarily set as 100. The results presented are mean ± SD from three experiments.

Given that not every putative ORF found in the sequenced genome is expressed, we performed real-time PCR to determine the expression levels of KLIKK proteases in *in vitro* culture of a laboratory strain of *T. forsythia* ATCC 43037 (Figure 3). We were able to detect transcripts for each KLIKK protease. Karilysin exhibited the highest expression level, while expression of the serine proteases miropsin-1, mirolase, and miropsin-2 was 3-fold lower, and expression of the two metalloproteases, forsilysin and mirolysin, was 10-fold lower than that of karilysin.

In prokaryotes, there is a well-described phenomenon of negative selection leading to the removal of any unnecessary genes (Burke and Moran, 2011). Therefore, we determined the expression of the KLIKK proteases *in vivo* in the GCF collected from *T. forsythia* positive patients suffering from different forms of periodontitis. Transcripts of all the KLIKK proteases were found in the majority of samples testing positive for the presence of *T. forsythia* (Table 1).

**Table 1:** **Prevalence of KLIKK protease transcripts in GCF from patients suffering from periodontitis**.

**Primers for:**	**Patients with chronic periodontitis: 37 samples**	**Patients with aggressive periodontitis: 17 samples**	**Healthy controls: 6 samples**
*T. forsythia*	28/37 (76%)	15/17 (88%)	1/6 (17%)
forsilysin	26/28 (93%)	11/15 (73%)	0/1 (0%)
miropsin-1	22/28 (79%)	9/15 (60%)	1/1 (0%)
mirolysin^*^	24/28 (86%)	13/15 (87%)	0/1 (0%)
mirolase	27/28 (96%)	15/15 (100%)	1/1 (100%)
miropsin-2	28/28 (100%)	15/15 (100%)	1/1 (100%)
karilysin^*^	27/28 (96%)	14/15 (93%)	1/1 (100%)

* *Results for karilysin and mirolysin were published previously (Jusko et al., 2012, under review)*.

### Proteolytic activity of the KLIKK proteases

**Figure 4:**
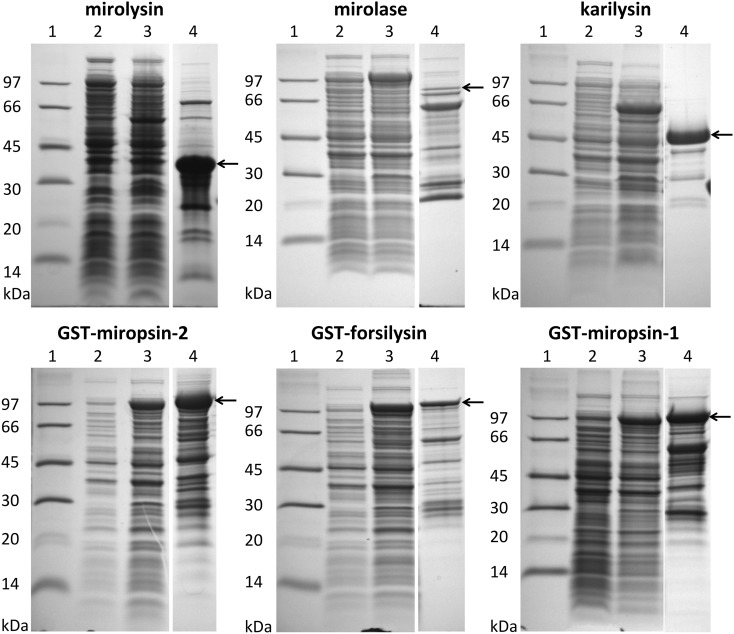
**Expression and purification of KLIKK proteases**. *Escherichia coli* extracts and fusion proteins or tag-free proteases after purification on glutathione-Sepharose and, if applicable, in column digestion with PreScission protease, were resolved by SDS-PAGE. Lane 1, protein molecular weight marker “LMW.” Lane 2, *E. coli* extracts before IPTG induction. Lane 3, *E. coli* extracts at 6 h after protein expression stimulation with IPTG. Lane 4, purified proteases as tag-free proteolytic enzymes or fusion proteins with GST. The arrows indicate the band corresponding to the theoretical molecular mass of the purified protein.

To confirm that the KLIKK proteases are active proteolytic enzymes, we expressed each of them as fusion proteins with an N-terminal glutathione-S-transferase (GST). The proteases were purified by affinity chromatography on glutathione-Sepharose (Figure 4). For the recombinant mirolysin, mirolase, and karilysin, the GST was removed by cleavage with the PreScission protease. The activities of miropsin-1, miropsin-2, and forsilysin were tested as fusion proteins due to their instability without the GST tag. In the case of mirolysin, despite using different *E. coli* strains and screening many expression conditions, we were unable to obtain a sufficient amount of the full-length enzyme. For this reason, we expressed this protease without the CTE region, but with the NTP, which could act as a chaperone and, thus, be required for proper folding of the enzyme (Bryan, 2002) (Figure 2). The recombinant proteins were used in further experiments, as described below.

**Figure 5:**
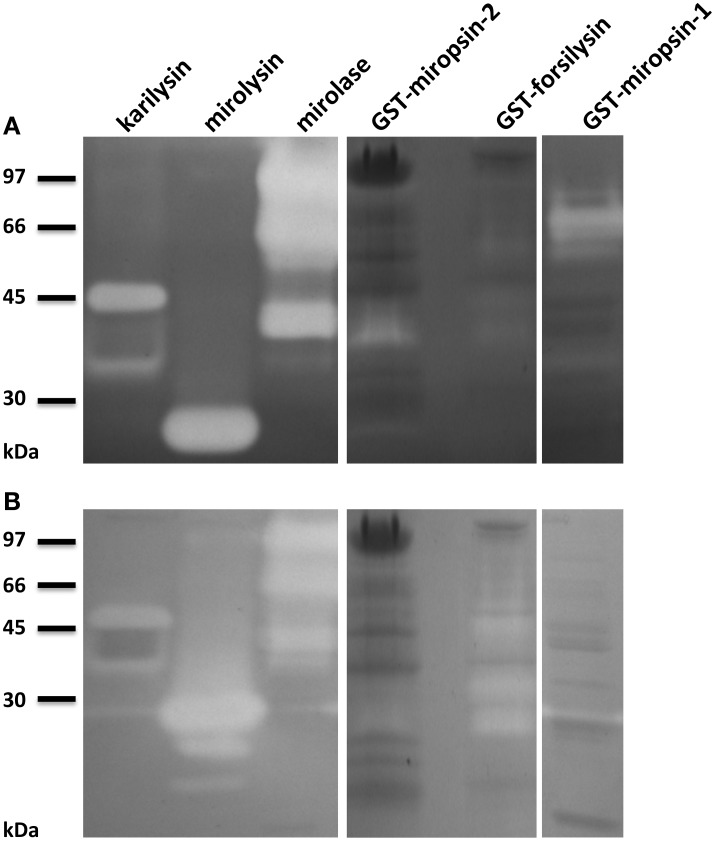
**Zymography analysis of KLIKK proteases**. The activity of six proteases (karilysin (2.5 μg), mirolysin (2.5 μg), mirolase (2.5 μg), GST-miropsin-2 (15 μg), GST-forsilysin (5 μg), and GST-miropsin-1 (7.5 μg)) was analyzed by zymography using casein **(A)** and gelatin **(B)** as a substrate.

First, activity of the purified KLIKK proteases was tested in zymography with two substrates, gelatin, and casein (Figure 5). All investigated proteases were active against at least one substrate. Moreover, in all cases, with the exception of miropsin-2, there was more than one band of activity visible. This observation indicates that the KLIKK proteases, similarly to karilysin, undergo auto-processing into lower molecular mass forms.

**Figure 6:**
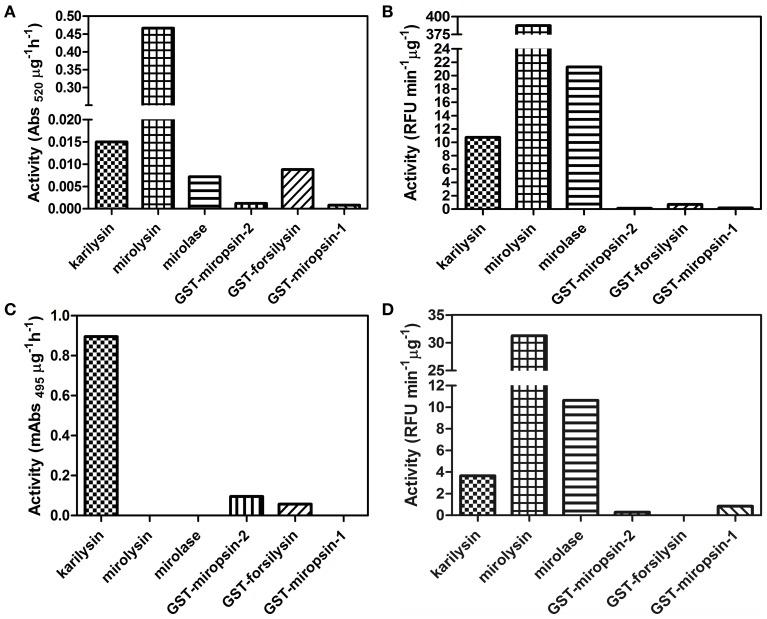
**Activity of KLIKK proteases against four protein substrates: Azocoll (A), DQ-gelatin (B), Elastin Congo Red (C), and FTC-casein (D)**. For Elastin Congo Red, human neutrophil elastase was used as a positive control, and its activity in the same conditions were 3.8 mAbs_495_ μg^−1^ h^−1^.

Finding a synthetic substrate for every novel protease paves the way for further research, so we checked whether the KLIKK proteases are active against the commercially available labeled protein substrates Azocoll, DQ-gelatin, Elastin Congo Red, and FTC-casein (Figure 6). For three proteases, forsilysin, mirolysin, and mirolase, we identified at least one substrate that was digested with efficiency comparable to that of karilysin. Although the activity of the two remaining proteases, miropsin-1 and miropsin-2, were several fold lower than karilysin, we were able to identify substrates that could be used for monitoring the proteolytic activity. The low activity is most likely due to enzyme latency imposed by the NTP and/or a low level of auto-activation in the presence of the GST tag. The most active enzyme against the investigated substrates was mirolysin. Interestingly, three proteases, karilysin, forsilysin, and miropsin-2, were able to digest Elastin Congo Red. Elastin is one of the major components of the connective tissue. Since miropsin-1 and miropsin-2 exhibited low activity to the protein substrates, we examined whether these two serine proteases were active against several synthetic amino acid chromogenic substrates with p-nitroaniline (*p*NA) as a leaving group including MeoSuc-AAV-*p*NA, Suc-AAPF-*p*NA, Suc-AAPL-*p*NA, Suc-AAPA-*p*NA, p-Tosyl-GPK-*p*NA, and Suc-AAPR-*p*NA. Miropsin-2 was active against one substrate, Suc-AAPL-*p*NA and the activity was 15 mAbs_410_ μg^−1^ h^−1^.

## Discussion

An annotated *T. forsythia* genome is currently available (GenBank database, accession number: CP003191), but initial attempts to amplify some of the genes encoding the KLIKK proteases failed. Also a previous attempt to amplify the karilysin gene failed due to an incorrect sequence in the database (Karim et al., 2010). Therefore, we re-sequenced parts of the genome encompassing genes encoding the putative KLIKK proteases and found that the available genomic sequence contained a number of errors. The sequences of the *T. forsythia* genome fragments encoding the KLIKK proteases may have generated problems in the correct assembly of the genome due to the high A/T content, the presence of A/T tracks, and the identical sequential motifs shared by the KLIKK proteases (Schatz et al., 2010).

The corrected sequences revealed that each KLIKK protease gene is preceded by a 50 bp A/T rich tract, which is probably responsible for the initiation of protein translation through a non-classical, Shine–Dalgarno sequence independent mechanism. Similar phenomena were described for other members of the *Bacteroidetes* (Accetto and Avguštin, 2011), as well as *E. coli* (Skorski et al., 2006; Nakagawa et al., 2010). The non-classical mechanism depends on the presence of a secondary structure of low stability (ΔG > −2.1 kcal mol^−1^) in a 5′UTR region adjacent to a start codon (Accetto and Avguštin, 2011). The analysis of a 30 bp long 5′UTR region of KLIKK proteases by mfold RNA (Zuker, 2003) revealed the presence of very weak secondary structures (ΔG > 0.4 kcal mol^−1^). Moreover, a Shine–Dalgarno sequence could not be identified, suggesting that all of the KLIKK proteases use a Shine–Dalgarno sequence independent mechanism for initiation of translation.

The bioinformatics analysis revealed that the KLIKK proteases possess a unique multi-domain structure. Briefly, the CD is flanked by the NTP and CTE domains, which do not have homology to any known proteins. The length of NTP is unique for each protease and varies from 14 (karilysin) to 225 residues (forsilysin and miropsin-1). The results obtained for karilysin suggest that the NTP region is responsible for the latency of the KLIKK proteases. Due to the irreversible nature of peptide bond hydrolysis, proteases are often synthesized as inactive zymogens to prevent inappropriate proteolytic damage. The zymogenicity is frequently exerted by the NTP, which needs to be removed to generate a full active protease. In Gram-negative bacteria, this mechanism is thought to protect the periplasm against unwanted proteolytic activity (Veillard et al., 2013).

The CD is followed by a CTE, which ends in a conserved region of 86 residues with some similarity to the CTD found in many secretory proteins of *P. gingivalis* and *T. forsythia* (Nguyen et al., 2007; Veith et al., 2009). The CTD is a signal for secretion of proteins through a recently described type 9 secretion system (T9SS). The 22 C-terminal residues of the CTD are essential for targeting proteins to the outer membrane translocon (Shoji et al., 2011). Interestingly, among all identified proteins terminating with a CTD in *T. forsythia*, only a few amino acids residues within the essential region are highly conserved, PxGxYVV and KxxxK (marked in red in Figure 2). The sequence of the rest of the CTD is variable (Veith et al., 2013). Therefore, the virtually identical CTDs in all the KLIKK proteases are unusual, and may indicate a common origin of this domain, which was acquired relatively recently. Explanation of the origin and biological impact of the CTD requires further research.

By analogy to *P. gingivalis*, the presence of the CTD fragment implies that the KLIKK proteases are secreted, post-translationally modified, and then retained on the bacterial surface. This assumption is partially confirmed by the fact that karilysin is released into the medium in the form of the fully processed 18 kDa protease by the laboratory strain *T. forsythia* ATCC 43037 when grown under normal conditions (data not shown). However, the KLIKK proteases do not seem to be associated with the cell envelope, because none of them was found among 221 proteins identified in the cell envelope proteome of *T. forsythia* (Veith et al., 2009). By contrast, three KLIKK proteases, miropsin-2 (*bfor_c_1_10600*), karilysin (*bfor_c_1_10593*), and forsilysin (*bfor_c_1_14540*), were recently shown to be secreted by T9SS (Narita et al., 2014). Together, these findings fully confirmed the release of the KLIKK proteases as soluble forms into the extracellular environment.

Karilysin, the first thoroughly characterized protease of *T. forsythia*, processes itself into shorter forms resulting in formation of the mature enzyme, Kly18. This modification is accompanied by a large increase in proteolytic activity (Karim et al., 2010). Similarly, all KLIKK proteases, with the exception of miropsin-2, occur in several active forms, strongly suggesting that these KLIKK proteases also process themselves into low molecular weight forms through sequential proteolytic cleavages. Based on the results obtained for karilysin and mirolase, the final products of auto-processing of the KLIKK proteases is predicted to be CDs without the NTP or the CTE, and at least transiently accompanied by protease-resistant domains derived from the CTE (Karim et al., 2010; Ksiazek et al., 2015a). Thus, it is tempting to speculate that the variable region in the KLIKK proteases could have a biological function unique for each KLIKK protease. Solving the role of these domains in *T. forsythia* virulence will require further studies.

Recently, a genome of the non-pathogenic oral bacterium *Tannerella BU063* (oral taxon 286), which is closely related to *T. forsythia*, was sequenced (Beall et al., 2014). None of the KLIKK homologs were identified in this genome. However, a *BU063* predicted ORF, *T229_10715* (GenBank accession number: ETK04104.1), shows a high degree of similarity to the CD of mirolase, but lacks a SP, NTP, and the variable region characteristic of the CTE in the KLIKK proteases. Interestingly, T229_10715 possesses a predicted CTD, which is significantly different from the CTD conserved in the KLIKK proteases. These findings may suggest that during evolution, virulence genes encoding proteases already present in the ancestral genome, or acquired through horizontal gene transfer (Cerdà-Costa et al., 2011), were fused to sequences coding for the SP, NTE, and CTE, thus generating the KLIKK proteases. The change in destination of the proteases from cytoplasm to extracellular environment would allow for acquisition of novel pathogenesis-related functions. Interestingly, *T. forsythia* also produces a protease inhibitor of the serpin superfamily, miropin (Ksiazek et al., 2015b), with the likely function of preventing proteolytic damage from secreted serine proteases. A miropin homolog (GenBank accession number: ETK05009.1) is present in *Tannerella BU063*, which possesses genes encoding putative serine proteases related to the *T. forsythia* KLIKK proteases. By stark contrast, neither the KLIKK proteases nor the miropin homologs are present in the sequenced genome of *Tannerella sp. 6_1_58FAA_* CT1 isolated from the human gastrointestinal tract (GenBank accession number: NZ_ACWX00000000.1). Thus, acquisition of the KLIKK proteases may be associated with the gain of virulence by *T. forsythia*.

Collectively, our data indicate that genes of the KLIKK proteases are efficiently transcribed in *in vitro* culture. Moreover, transcripts of all the KLIKK proteases were detected in GCF sampled from periodontitis sites infected with *T. forsythia*. This observation suggests an active role for the KLIKK proteases in periodontal lesions. This hypothesis is corroborated by the involvement of karilysin in evasion of innate host defenses through cleavage of LL-37 (Koziel et al., 2010) and inactivation of the complement system by karilysin and mirolysin (Jusko et al., 2012; Jusko, unpublished). However, the elastin degradation by three of the KLIKK proteases described here may also contribute to the connective tissue damage at the infected periodontal site. Although the exact biological functions of the KLIKK proteases still requires further research (Van Damme et al., 2008), they do now appear to be potent and versatile virulence factors of *T. forsythia*.

## Author contributions

MK, DM, SE, IT designed and conceived experiments; MK, DM, SE, IT performed the experiments; MK, DM, SE, JE, JP analyzed data; MK, DM and JP wrote the paper.

### Conflict of interest statement

The authors declare that the research was conducted in the absence of any commercial or financial relationships that could be construed as a potential conflict of interest.

## Abbreviations

SP: signal peptide
NTP: N-terminal profragment
CD: catalytic domain
CTE: C-terminal extension
CTD: C-terminal domain.
