# Supplementary material for: KLIKK proteases of Tannerella forsythia: putative virulence factors with a unique domain structure
Source: Front Microbiol. 2015 Apr 21;6:312. doi: 10.3389/fmicb.2015.00312 (PMC4404884; doi:10.3389/fmicb.2015.00312)
Supplement: Supplementary file 1 [file Table1.DOC]

***Supplementary Material***

**KLIKK proteases of *Tannerella forsythia*: putative virulence factors with a unique domain structure**

**Miroslaw Ksiazek^1†,*^, Danuta Mizgalska^1†,*^, Sigrum Eick^2^, Ida B. Thøgersen^3^, Jan J. Enghild^3^, Jan Potempa^1,4,*^**

^1^Faculty of Biochemistry, Biophysics and Biotechnology, Department of Microbiology, Jagiellonian University, Krakow, Poland

^2^Laboratory of Oral Microbiology, Department of Periodontology, University of Bern, Bern, Switzerland

^3^Center for Insoluble Protein Structures (inSPIN) and Interdisciplinary Nanoscience Center (iNANO) at the Department of Molecular Biology and Genetics, Aarhus University, Aarhus, Denmark

^4^Department of Oral Immunology and Infectious Disease, University of Louisville School of Dentistry, Louisville, KY, USA

^†^These authors contribute equally and share first authorship.

***Correspondence:**

Miroslaw Ksiazek, Danuta Mizgalska and Jan Potempa,

Department of Microbiology

Faculty of Biochemistry, Biophysics and Biotechnology

Jagiellonian University

ul. Gronostajowa 7

30-387 Krakow, Poland

E-mails: [ksiazek.miroslaw@gmail.com](mailto:ksiazek.miroslaw@gmail.com), dankamizgalska@gmail.com, jan.potempa@louisville.edu

**Supplementary Table 1:** **Primers used in the study.**

| **Molecular cloning** | | | | |
| --- | --- | --- | --- | --- |
| Primer’s name | Sequence (5’- … - 3’)  (restriction sites are underlined, name of restriction enzyme in bracket) | | | |
| BFO_2661F | ACAGGATCCTCTGAGTTGAATATGGAAC (BamhI) | | | |
| BFO_2661R | CGGCTCGAGTTACTTCTTAATCAATTTCTGC (XhoI) | | | |
| BFO_2679F | CTTGGATCCCAAGTAGAGACAAGGTTCTTTCC (BamHI) | | | |
| BFO_2679R | TCACTCGAGTTACTTTTTAATCAACTTCTGCGTA (XhoI) | | | |
| BFO_1668F | TTCGGATCCCAAAAAACCATTCTTCGAA (BamHI) | | | |
| BFO_1668R | TCTCTCGAGTTATTTTTTGATCAACTTCTGCG (XhoI) | | | |
| BFO_1679F | GTCGGATCCCAAATAACGACCCATGAAGTTCC (BamHI) | | | |
| BFO_1679R | CGTGAATTCTTATTTTTTGATCAACTTCTGCGTATACGTC (EcoRI) | | | |
| **Real time PCR** | | | | |
| Gene | Primer’s name | Sequence (5’- … - 3’) | Annealing  Temp (^o^C) | |
| *BFO_2661* | 341fqRT  341rqRT | CTCGTAGTGTGCCTTCTTCCAC  GCCTGATCGGCATTCATTCGG | 55 | |
| *BFO_2665* | 347fqRT  347rqRT | GAATACATCCGTAAGAGTAGCGG  AGGGCTGTCATTGTCGTTCGG | 56 | |
| *BFO_2679* | 364seqF2  364rqRT | ATCCAACGAAGAAGGAGATATGC  CCGAACTTCCTATCTGACCG | 56 | |
| *BFO_2683* | 367f2  367rqRT | GGGAATCATGGTGACGGATATCC  GATGACCGATCTCATGTGCCG | 56 | |
| *BFO_1668* | qPCR2162F  qPCR2162R | GAGTCTGCGAATGACTGAACCAG  CTGTAATGCCCACTGCACGTCC | 55 | |
| *BFO_1679* | 14527qRT2F  14527qRT2R | ACGATAAGTTCTTGTTGCCT  GCACCGTTTGATTCGTTATT | 55 | |
| **Determination of KLIKK proteases expression *in vivo*** | | | | |
| Gene | Primer’s name | Sequence (5’- … - 3’) | Annealing  Temp (^o^C) | |
| *T. forsythia* ATCC43037 | Tf-1  Tf-2 | GCGTATGTAACCTGCCCGCA  TGCTTCAGTGTCAGTTATACCT | 60 | |
| *BFO_2665* | tfslp-1  tfslp-2 | TGCCGCAAATCATAATGGTA  GTCCATCCCTTCCTTGAGTG | 58 | |
| *BFO_2679* | tftlp-1  tftlp-2 | AAGAAGGAGATATGCTTTATGGTC TCCGACTCTTTACTCCAAACAG | 56 | |
| *BFO_1668* | tfthb-1  tfthb-2 | GATGATGGGTTTACAATTGACG  TCTAGTAATTTGTTCTCCAATTTGC | 56 | |
| *BFO_1679* | tftlb-1  tftlb-2 | TCGCATCGACTATGGATACAG  GATTGCTGATCGCATCGAA | 57 | |
| **Resequencing of *loci* encoding putative proteases with KLIKK ending** | | | | |
| Primer’s name | Sequence (5’- … - 3’) | | | Usage |
| Region between BFO_1668 and BFO_1679 with surroundings | | | | |
| 74F | GGCAACTTTTAGGTCCTGGCAAC | | | PCR |
| 62R | GCTTCGTGTCCGGTAATAGAAGGC | | | PCR |
| 74seqF | GAGTTTCTTACGGCAAATTCAGTG | | | Seq |
| 62seqR | GGAAATTATATCCGGCTCCATC | | | Seq |
| 74seq2F | GGATATCCTTGTTATATACCTTTGG | | | Seq |
| 62seq2R | GTTTATACTTGTTTCCGCGTTCC | | | Seq |
| 74seqFR | CACTGAATTTGCCGTAAGAAACTC | | | Seq |
| 62seqRR | GATGGAGCCGGATATAATTTCC | | | Seq |
| 62seq3R | GATGTTCATATCGGCGTTATGAC | | | Seq |
| 74dR | CACATAGAACTTTGCTCCTTTAGGC | | | PCR |
| purDF | GCCTTTACTCCCTTCCAGTTGAGC | | | PCR |
| 74dseqR | CCTTTAGCCTCCACAATTTGC | | | Seq |
| purseqR | TCGTAGTCCTTGTTCTTCTCG | | | Seq |
| 62dF | CGTATCGGTATCTCATCAAGGTGG | | | PCR |
| yienR | GAACCGTCTCTGCTGCGTTATGTG | | | PCR |
| 62dseqF | GGAAGGACGAGCAAGAATTAATG | | | Seq |
| yienseqF | GTCAATGGTTATACGTGGTACAG | | | Seq |
| BFO_0703 | | | | |
| DM19f | GTGATCTTGGATGGAAGTTATGTC | | | PCR, Seq |
| DM20r | GCTTCGACTTGATGTCGAAGTAC | | | PCR, Seq |
| DM21f | CCAAGGAGAGATGGTGATTG | | | Seq |
| Region between BFO_2661 and BF0_2683 with surroundings | | | | |
| 364P1F | TGCCTCAAACTCTCGAAGGTC | | | PCR |
| 364P1R | GGAATGACAGGGGTATATGTTGTAGATAC | | | PCR |
| 364s1F | GTCATCCTGAAGAATCGATCG | | | Seq |
| 364s1R | CATCAGCTTCCATCCAACAATC | | | Seq |
| 364s2F | GTATTCAACGATTTCTACTTGCCC | | | Seq |
| 364s2R | TCCTGTGATTTGTACCGTGATC | | | Seq |
| DM1f | GTATTCTACTCCGACAATTACTTCG | | | PCR |
| DM2r | CTTTCTGTAGTCGTCAAATGGGACG | | | Seq |
| DM3f | GGGAATCATGGTGACGGATATCC | | | Seq |
| DM4r | GCAAAACATCTCCACCATTG | | | Seq |
| DM5r | CGCCATATCCGGTGCAGATAGC | | | PCR |
| DM6f | ATCCAACGAAGAAGGAGATATGC | | | PCR |
| DM7f | TCCAACCGGTTGTCACATTC | | | Seq |
| DM8f | GAAGTGACTTTGCAGCTGAC | | | Seq |
| DM9r | GTAAGATCACCGCAAGGAAC | | | Seq |
| DM10r | CCTGCCGACCAATAATAGC | | | PCR, Seq |
| DM11f | CGGCAAGAGATTTAGGCTCTGCC | | | PCR |
| DM12f | GATGCTCATGCTGCCGTAGCC | | | Seq |
| DM13f | GGATGATAGCTGACTGCTTGA | | | Seq |
| DM14f | CTGACGCTCTGAATGACAAG | | | Seq |
| DM15r | GTGGAAGAAGGCACACTACGAG | | | Seq |
| DM16f | GGACAAGTAGAGCGTATGAGATCG | | | Seq |
| DM17f | CGAATGAGCCGACGTTCCAAATCC | | | Seq |
| DM18r | GATCGTCTATGTCGACAAGAACG | | | PCR |

PCR – primers used for amplification of genome fragment; Seq – primers used for sequencing.
